# Prognostic value of positive lymph node ratio, tumor deposit, and perineural invasion in advanced colorectal signet-ring cell carcinoma

**DOI:** 10.3389/fmolb.2025.1617787

**Published:** 2025-08-01

**Authors:** Liang Chu, Han Wang, Tao Ling, Shuhan Feng, Yucheng Ding, Yan Zhang, Ying Pan, Cenzhu Wang, Xiaohong Wang, Lei Liu

**Affiliations:** ^1^ Department of Surgery, The Affiliated Yixing Hospital of Jiangsu University, Yixing, Jiangsu, China; ^2^ Clinical Laboratory, Hainan Medical University, Haikou, Hainan, China; ^3^ Department of Gastroenterology, Jinhua Central Hospital Affiliated to Zhejiang University School of Medicine, Jinhua, China; ^4^ Department of Gastroenterology, The Affiliated Yixing Hospital of Jiangsu University, Yixing, Jiangsu, China; ^5^ Inpatient Management Center, Nanjing Drum Tower Hospital Affiliated to Nanjing University Medical School, Nanjing, China; ^6^ Department of Oncology, The Affiliated Wuxi People’s Hospital of Nanjing Medical University, Wuxi People’s Hospital, Wuxi Medical Center, Nanjing Medical University, Wuxi, China; ^7^ Department of Gastroenterology, XuZhou Central Hospital Affiliated to Medical School of Southeast University, XuZhou, China

**Keywords:** colorectal signet ring cell carcinoma, lymph node ratio, tumor deposit, perineural invasion, prognosis

## Abstract

**Background:**

The aim of this study was to assess the prognostic significance of positive lymph node ratio (LNR), tumor deposits (TD), and perineural invasion (PNI) in advanced colorectal signet-ring cell carcinoma (SRCC).

**Methods:**

A multicenter retrospective cohort analysis was conducted involving 677 patients with advanced colorectal SRCC. The associations of variables with CSS and OS were analyzed using the Kaplan-Meier method and multivariable Cox proportional hazards models. A nomogram model was developed to predict outcomes.

**Results:**

High-LNR, TD-positive, and PNI-positive were associated with poorer CSS and OS in both the training and validation cohorts. Multivariate Cox analysis identified T stage, M stage, TD, CEA, chemotherapy, and LNR as independent prognostic factors. A prognostic nomogram model incorporating these variables demonstrated excellent calibration and satisfactory predictive accuracy. Survival curves generated from individualized nomogram scores effectively discriminated prognostic outcomes (*P* < 0.001). The combined variable of LNR, TD, and PNI significantly enhanced the predictive performance. Specifically, the combined variable exhibited the highest relative contribution to OS at 23.4%, surpassing that of T and M stages. For CSS, its relative contribution was 21.4%, ranking second only to T and M stages.

**Conclusion:**

LNR, TD, and PNI served as prognostic factors for advanced colorectal SRCC. The combined analysis demonstrated a higher prognostic predictive value.

## Introduction

Signet ring cell carcinoma (SRCC) was a distinct subtype of adenocarcinoma, showing remarkable differences from adenocarcinoma in pathological features, clinical behavior and prognosis (Allart et al., 2022). It was characterized by abundant intracytoplasmic mucin that pushes the nucleus to one side, giving the cells a signet-ring-like appearance (Anthony et al., 1996). The diagnostic criterion for SRCC was the presence of more than 50% signet ring cells in the tumor tissue (Nagtegaal et al., 2020). Compared to colorectal conventional adenocarcinoma, colorectal SRCC tended to occur in younger individuals, was often diagnosed at an advanced stage, and had a lower survival rate (Weng et al., 2022; Korphaisarn et al., 2019). Notably, in a study involving 173,460 CRC patients, 1,932 (1.11%) were diagnosed with colorectal SRCC, and 76.04% of these cases were at stage III - IV (Shi et al., 2019). Studies have shown that SRCC was characterized by a high frequency of mutations in epithelial-mesenchymal transition (EMT)- and stemness-related genes (such as RNF43, CDH1, and SMAD4), accompanied by excessive activation of the transforming growth factor-β (TGF-β) pathway (An et al., 2021). Meanwhile, upregulation of the autophagic pathway and enrichment of immune-related proteins further promoted tumor progression (Chaudhary et al., 2025). These altered molecular features, combined with tumor stemness regulation and changes in the immune microenvironment (Ye et al., 2024a; Ye et al., 2024b; Ye et al., 2024c; Ye et al., 2025), collectively lead to poor response to conventional therapeutic regimens (Pande et al., 2008).

In current clinical practice, prognosis of CRC was primarily assessed using the TNM staging system (Weiser, 2018). However, this system did not account for the number of lymph nodes retrieved during surgery, potentially resulting in an underestimation of the actual number of metastatic lymph nodes and subsequent downstaging (Berger et al., 2005). The lymph node ratio (LNR) was defined as the ratio of positive lymph nodes to the total number of lymph nodes retrieved and may exhibit superior performance in predicting prognosis (Gartagani et al., 2022). Tumor deposits (TD) served as a supplement to TNM staging in CRC. When tumor deposits were present but there was no lymph node metastasis, they were considered as N1c stage (Brierley et al., 2017). Perineural invasion (PNI) refers to cancer cells invading nerves, around nerves, or directly through them, involving at least 33% of the nerve’s circumference or any layer of the nerve sheath structure (Zhang B. et al., 2023).

Research on advanced colorectal SRCC remains limited. Previous studies predominantly relied on case reports or small sample analyses. By leveraging multicenter data, this study systematically evaluated the prognostic value of LNR, TD, and PNI in advanced colorectal SRCC. Furthermore, a combined analysis was performed to develop a clinically applicable prognostic model.

## Patients and methods

### Patients

The study included colorectal SRCC patients from the SEER database between 2011 and 2018 as the training cohort. Concurrently, colorectal SRCC patients from four tertiary hospitals (the Second Affiliated Hospital of Nanjing Medical University, Yixing People’s Hospital, Drum Tower Hospital Affiliated to Nanjing University School of Medicine, and Xuzhou Central Hospital) in China were included as the external validation cohort. The inclusion criteria were as follows: (1) Patients with stage III or stage IV; (2) Individuals who underwent surgical treatment. Exclusion criteria included: (1) Non-primary tumors or surgeries performed at non-primary sites; (2) Concurrent presence of other tumors; (3) Perioperative death (≤1 month post-surgery); (4) Incomplete clinical or pathological data.

### Study variables

Baseline data including demographic characteristics, tumor size, tumor site, grade, TNM staging, TD, LNR, PNI, carcinoembryonic antigen (CEA) levels, and chemotherapy status were collected. Survival outcomes for patients in the SEER dataset were obtained through systematic queries, while survival outcomes in Chinese hospitals were obtained through telephone follow-ups and death registry queries.

The primary outcome of the study was cancer-specific survival (CSS), defined as the time from diagnosis to death due to colorectal SRCC. The secondary outcome was overall survival (OS), defined as the time from diagnosis to death from any cause. This study adhered to the Helsinki Declaration. Ethical approval was obtained from the institutional review boards of the four tertiary Chinese hospitals with approval numbers: IRB Approval No. 2020-092, IRB Approval No. 2022-158, IRB Approval No. 2022-469-02, and IRB Approval No. XZXY-LK-20240116-007.

### Statistical methods

Categorical variables were presented as counts (%) and compared using Pearson’s chi-square test or Fisher’s exact test. LNR was categorized into tertiles using X-tile software, which was identified by scoring the maximum X-squared value in the Kaplan-Meier test based on survival time and outcomes (Tang et al., 2018). Survival curves were constructed using the Kaplan-Meier method, and both univariate and multivariate prognostic analyses were carried out by Cox proportional hazards model. Variables that exhibited statistical significance in the training cohort were subsequently incorporated into the nomogram model. Internal validation was carried out through bootstrapping, and external validation was performed in four Chinese cohort. Model accuracy was assessed using receiver operating characteristic curves (ROC), while calibration curves were employed to evaluate the consistency between the predicted probabilities and the observed probabilities. Relative variable contributions to prognosis were calculated in R. All statistical tests were two-sided, with *p* < 0.05 considered significant. Statistical analyses were performed using SPSS 22.0, GraphPad Prism 9.4.1, and R 4.3.1 software.

## Results

### Clinical and pathological characteristics of patients

**FIGURE 1:**
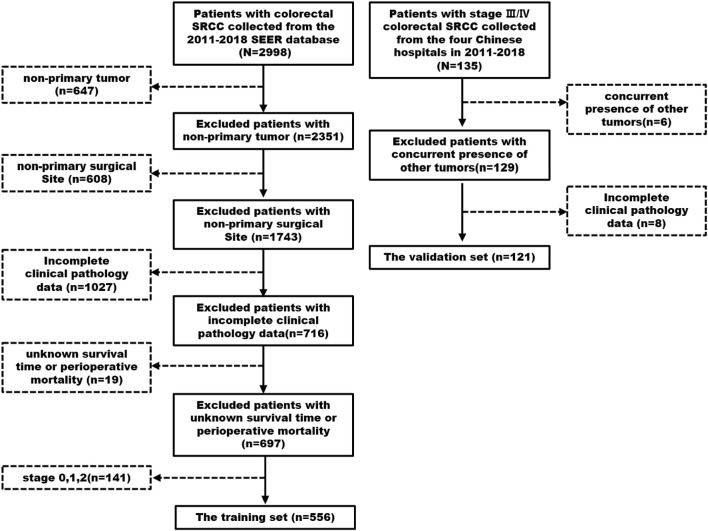
Flowchart of the study.

A total of 677 patients were included in the study, with 556 in the training cohort and 121 in the validation cohort, as illustrated in the flowchart (Figure 1). X-tile software categorized LNR into three groups: low-LNR (LNR <0.3), moderate-LNR (0.3 ≤ LNR ≤0.7), and high-LNR (LNR >0.7) (Supplementary Figure S1). In the training cohort, high-LNR, TD-positive, and PNI-positive patients were associated with T4 stage and >4 metastatic lymph nodes (Table 1). In the validation cohort, high-LNR and PNI-positive patients frequently had more than four metastatic lymph nodes, while TD-positive patients tended to exhibit PNI and distant metastasis (Table 2).

**TABLE 1:** Patient characteristics by LNR, TD and PNI in the training cohort.

	Low-LNR (N = 210)	Moderate-LNR (N = 193)	High-LNR (N = 153)	*p* value	TD-negative (N = 274)	TD-positive (N = 282)	*p value*	PNI-negative (N = 330)	PNI-positive (N = 226)	*p value*
Gender				0.08			0.13			0.66
Female	102 (48.57)	92 (47.67)	90 (58.82)		131 (47.8)	153 (54.3)		166 (50.3)	118 (52.2)	
Male	108 (51.43)	101 (52.33)	63 (41.18)		143 (52.2)	12,945.7 ()		164 (49.7)	108 (47.8)	
Age				0.17		<0.01	<0.01		0.02	0.09
Media (Q1-Q3)	67.50 (54.00–78.25)	63.00 (52.00–75.00)	64.00 (53.00–74.00)		68 (55.0–80.0)	63 (51.0–73.25)		67 (55.0–77.0)	62 (50.0–74.0)	
Tumor size				0.02			0.02			0.42
≤5 cm	100 (47.62)	90 (46.63)	52 (33.99)		133 (48.5)	109 (38.7)		139 (42.1)	103 (45.6)	
>5 cm	110 (52.38)	103 (53.37)	101 (66.01)		141 (51.5)	173 (61.3)		191 (57.9)	123 (54.4)	
Tumor site				<0.01			0.02			0.23
Right	152 (72.38)	126 (65.29)	79 (51.63)		192 (70.1)	165 (58.5)		220 (66.7)	137 (60.6)	
Left	43 (20.48)	52 (26.94)	58 (37.91)		63 (23.0)	90 (31.9)		82 (24.8)	71 (31.4)	
Rectum	15 (7.14)	15 (7.77)	16 (10.46)		19 (6.9)	27 (9.6)		28 (8.5)	18 (8.0)	
Grade				0.01			0.04			0.08
Moderate	18 (8.57)	7 (3.63)	3 (1.96)		19 (6.9)	9 (3.2)		21 (6.4)	7 (3.1)	
Poor	192 (91.43)	186 (96.37)	150 (98.04)		255 (93.1)	273 (96.8)		309 (93.6)	219 (96.9)	
T stage				<0.01			<0.01			<0.01
T1-3	115 (54.76)	89 (46.11)	47 (30.72)		161 (58.8)	90 (31.9)		179 (54.2)	72 (31.9)	
T4	95 (45.24)	104 (53.89)	106 (69.28)		113 (41.2)	192 (68.1)		151 (45.8)	154 (68.1)	
N stage				<0.01			<0.01			<0.01
<4 nodes	132 (62.86)	9 (4.66)	3 (1.96)		98 (35.8)	46 (16.3)		105 (31.8)	39 (17.3)	
≥4 nodes	78 (37.14)	184 (95.34)	150 (98.04)		176 (64.2)	236 (83.7)		225 (68.2)	187 (82.7)	
M stage				<0.01			<0.01			<0.01
M0	169 (80.48)	123 (63.73)	76 (49.67)		217 (79.2)	151 (53.5)		240 (72.7)	128 (56.6)	
M1	41 (19.52)	70 (36.27)	77 (50.33)		57 (20.8)	131 (46.5)		90 (27.3)	98 (43.4)	
TD				<0.01						<0.01
Absent	139 (66.19)	90 (46.63)	45 (29.41)		NA	NA		198 (60.0)	76 (33.6)	
Present	71 (33.81)	103 (53.37)	108 (70.59)		NA	NA		132 (40.0)	150 (66.4)	
PNI				<0.01			<0.01			
Absent	149 (70.95)	106 (54.92)	75 (49.02)		198 (72.3)	132 (46.8)		NA	NA	
Present	61 (29.05)	87 (45.08)	78 (50.98)		76 (27.7)	150 (53.2)		NA	NA	
CEA				0.02			0.10			0.85
Negative	99 (47.14)	87 (45.08)	50 (32.68)		126 (46.0)	110 (39.0)		139 (42.1)	97 (42.9)	
Positive	111 (52.86)	106 (54.92)	103 (67.32)		148 (54.0)	172 (61.0)		191 (57.9)	129 (57.1)	
Neoadjuvant therapy				0.95			0.14			0.13
No	197 (93.81)	180 (93.26)	144 (94.11)		261 (95.3)	260 (92.2)		305 (92.4)	216 (95.6)	
Yes	13 (6.19)	13 (6.74)	9 (5.88)		13 (4.7)	22 (7.8)		25 (7.6)	10 (4.4)	
Chemotherapy				0.66			0.11			0.98
No	69 (32.86)	56 (29.02)	50 (32.68)		95 (34.7)	80 (28.4)		104 (31.5)	71 (31.4)	
Yes	141 (67.14)	137 (70.98)	103 (67.32)		179 (65.3)	202 (71.6)		226 (68.5)	155 (68.6)	
LNR							<0.01			<0.01
Low	NA	NA	NA		139 (50.7)	71 (25.2)		149 (45.2)	61 (27.0)	
Moderate	NA	NA	NA		90 (32.8)	103 (36.5)		106 (32.1)	87 (38.5)	
High	NA	NA	NA		45 (16.4)	108 (38.3)		75 (22.7)	78 (34.5)	

TD, tumor deposit; PNI, perineural invasion; CEA, carcinoembryonic antigen; LNR, positive lymph node ratio.

**TABLE 2:** Patient characteristics by LNR, TD and PNI in validation cohort.

	Low-LNR (N = 36)	Moderate-LNR (N = 48)	High-LNR (N = 37)	*p value*	TD-negative (N = 72)	TD-positive (N = 49)	*p value*	PNI-negative (N = 65)	PNI-positive (N = 56)	*p value*
Gender				0.83			0.48			0.37
Female	24 (66.67)	29 (60.42)	24 (64.86)		44 (61.1)	33 (67.3)		39 (60.0)	38 (67.9)	
Male	12 (33.33)	19 (39.58)	13 (35.14)		28 (38.9)	16 (32.7)		26 (40.0)	18 (32.1)	
Age				0.94			0.71			0.76
Media (Q1-Q3)	58.50 (41.00–68.00)	59.00 (47.25–66.00)	57.00 (44.50–69.00)		57.5 (44.25–67.75)	60.0 (44.0–68.0)		59 (48.5–67.5)	59 (42.5–68.0)	
Tumor size				0.83			0.97			0.02
≤5 cm	17 (47.22	25 (52.01)	20 (54.05)		37 (51.4)	25 (51.0)		27 (41.5)	35 (62.5)	
>5 cm	19 (52.78)	23 (47.92)	17 (45.95)		35 (48.6)	24 (49.0)		38 (58.5)	21 (37.5)	
Tumor site				0.23			0.13			0.40
Right	6 (16.67)	16 (33.33)	11 (29.73)		20 (27.8)	13 (26.5)		21 (32.3)	12 (21.4)	
Left	9 (25.0)	11 (22.92)	13 (35.14)		15 (20.8)	18 (36.7)		16 (24.6)	17 (30.4)	
Rectum	21 (58.33)	21 (43.75)	13 (35.14)		37 (51.4)	18 (36.7)		28 (43.1)	27 (48.2)	
Grade				0.82			0.15			0.12
Moderate	1 (2.78)	1 (2.08)	2 (5.41)		4 (5.6)	0 (0.0)		4 (6.2)	0 (0.0)	
Poor	35 (97.22)	47 (97.92)	35 (94.59)		68 (94.4)	49 (100.0)		61 (93.8)	56 (100.0)	
T stage				0.86			0.15			0.14
T1-3	17 (47.22)	20 (41.67)	17 (45.95)		36 (50.0)	18 (36.7)		33 (50.8)	21 (37.5)	
T4	19 (52.78)	28 (58.33)	20 (54.05)		36 (50.0)	31 (63.3)		32 (49.2)	35 (62.5)	
N stage				<0.01			0.77			0.03
<4 nodes	19 (52.78)	6 (12.50)	3 (8.11)		16 (22.2)	12 (24.5)		20 (30.8)	8 (14.3)	
≥4 nodes	17 (47.22)	42 (87.50)	34 (91.89)		56 (77.8)	37 (75.5)		45 (69.2)	48 (85.7)	
M stage				0.64			0.02			0.40
M0	27 (75.00)	33 (68.75)	24 (64.86)		56 (77.8)	28 (57.1)		43 (66.2)	41 (73.2)	
M1	9 (25.00)	15 (31.25)	13 (35.14)		16 (22.2)	21 (42.9)		22 (33.8)	15 (26.8)	
TD				0.28						0.02
Absent	25 (69.44)	28 (58.33)	19 (51.35)		NA	NA		45 (69.2)	27 (48.2)	
Present	11 (30.56)	20 (41.67)	18 (48.65)		NA	NA		20 (30.8)	29 (51.8)	
PNI				0.34			0.02			
Absent	23 (63.89)	24 (50.0)	18 (48.65)		45 (62.5)	20 (40.8)		NA	NA	
Present	13 (36.11)	24 (50.0)	19 (51.35)		27 (37.5)	29 (59.2)		NA	NA	
CEA				0.79			0.48			0.33
Negative	16 (44.44)	25 (52.08)	18 (48.65)		37 (51.4)	22 (44.9)		29 (44.6)	30 (53.6)	
Positive	20 (55.56)	23 (47.92)	19 (51.35)		35 (48.6)	27 (55.1)		36 (55.4)	26 (46.4)	
Neoadjuvant therapy				0.18		0.399	0.22		0.685	0.51
No	35 (97.22)	47 (97.92)	33 (89.19)		67 (93.1)	48 (98.0)		61 (93.8)	54 (96.4)	
Yes	1 (2.78)	1 (2.08)	4 (10.81)		5 (6.9)	1 (2.0)		4 (6.2)	2 (3.6)	
Chemotherapy				0.47			0.63			0.23
No	13 (36.11)	17 (35.42)	9 (24.32)		22 (30.6)	17 (34.7)		24 (36.9)	15 (26.8)	
Yes	23 (63.89)	31 (64.58)	28 (75.68)		50 (69.4)	32 (65.3)		41 (63.1)	41 (73.2)	
LNR							0.28			0.34
Low	NA	NA	NA		25 (34.7)	11 (22.4)		23 (35.4)	13 (23.2)	
Moderate	NA	NA	NA		28 (38.9)	20 (40.8)		24 (36.9)	24 (42.9)	
High	NA	NA	NA		19 (26.4)	18 (36.7)		18 (27.7)	19 (33.9)	

TD, tumor deposit; PNI, perineural invasion; CEA, carcinoembryonic antigen; LNR, positive lymph node ratio.

### Survival curves associated with the prognosis of LNR, TD, and PNI

**FIGURE 2:**
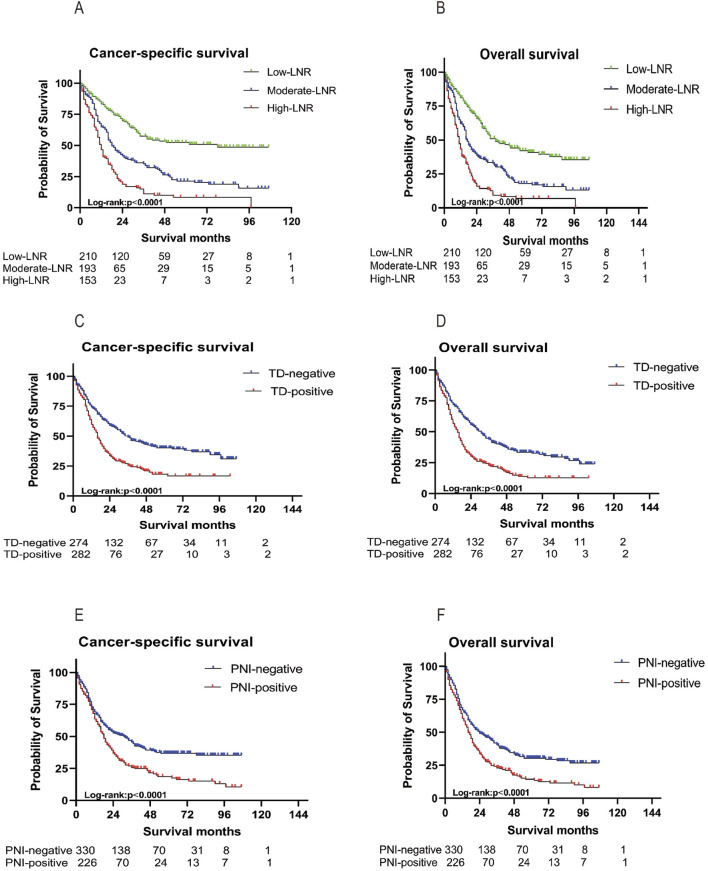
In the training cohort, Kaplan-Meier survival curves for CSS and OS based on LNR **(A, B)**, TD **(C, D)**, and PNI **(E, F)**. OS, Overall Survival; CSS, Cancer-Specific; LNR, positive lymph node ratio; TD, tumor deposit; PNI, perineural invasion.

Kaplan-Meier survival curves demonstrated that high-LNR, TD-positive, and PNI-positive were each associated with poorer CSS and OS in advanced colorectal SRCC in both cohorts (Figure 2; Supplementary Figure S2). This disparity was more pronounced in stage III tumors (Supplementary Figure S3). In stage IV tumors, only LNR remained significantly associated with survival, while TD and PNI showed no significance (Supplementary Figure S4). Similar trends were observed for OS. Interestingly, we conducted a combined analysis of TD, LNR, and PNI to construct a new combined variable, and patients were further classified into the triple-positive group (high LNR, TD-positive, PNI-positive), triple-negative group (low LNR, TD-negative, PNI-negative), or intermediate group. In the training cohort, 58 patients (10.4%) were triple-positive and 63 patients (11.3%) were triple-negative. The triple-positive group exhibited a higher cancer-related mortality risk compared to the triple-negative group, and this finding was validated in the validation cohort (Supplementary Figure S5).

### Univariate and multivariate analyses

Univariate analysis in the training cohort identified that tumor size, T stage, N stage, M stage, TD, PNI, CEA, chemotherapy, and LNR had prognostic value. Multivariate Cox model revealed T stage, M stage, TD, CEA, chemotherapy, and LNR as independent prognostic factors for CSS (Table 3). In the validation cohort, T stage, M stage, PNI, CEA, and LNR independently predicted CSS (Table 3). Similar results were observed for OS (Table 4). Notably, we integrated the combined analysis of TD, LNR, and PNI into a multivariate model that included tumor size, T stage, N stage, M stage, CEA, and chemotherapy. The results showed that the combined variable independently predicted both CSS and OS in the two cohorts (Table 5).

**TABLE 3:** Univariate and multivariate analyses of cancer-specific survival in the two cohorts.

	The training cohort				The validation cohort			
	Univariate		Multivariate		Univariate		Multivariate	
	HR (95% CI)	*p value*	HR (95% CI)	*p value*	HR (95% CI)	*p value*	HR (95% CI)	*p value*
Gender								
Female								
Male	0.90 (0.73–1.12)	0.35			0.78 (0.47–1.27)	0.32		
**Age**	**1.00 (1.00–1.01)**	**0.34**			**1.01 (0.99–1.02)**	**0.42**		
Tumor size								
≤5 cm								
>5 cm	1.47 (1.18–1.83)	<0.01	1.13 (0.91–1.42)	0.27	1.06 (0.67–1.69)	0.86		
**Tumor site**		**0.36**						
Right								
Left	0.99 (0.78–1.26)	0.96			1.04 (0.56–1.95)	0.90		
Rectum	0.74 (0.48–1.16)	0.16			0.96 (0.55–1.70)	0.90		
Grade								
Moderate								
Poor	1.17 (0.72–1.91)	0.52			1.84 (0.45–7.54)	0.39		
T stage								
T1-3								
T4	2.12 (1.69–2.65)	<0.01	1.57 (1.25–2.01)	<0.01	2.31 (1.40–3.82)	<0.01	1.69 (1.01–2.83)	0.05
N stage								
<4 nodes								
≥4 nodes	2.65 (1.98–3.54)	<0.01	1.26 (0.85–1.86)	0.26	1.55 (0.85–2.82)	0.16		
M stage								
M0								
M1	2.55 (2.06–3.17)	<0.01	1.65 (1.30–2.09)	<0.01	2.35 (1.47–3.76)	<0.01	2.32 (1.37–3.93)	<0.01
TD								
Absent								
Present	1.85 (1.49–2.29)	<0.01	1.30 (1.02–1.64)	0.03	2.46 (1.55–3.97)	<0.01	1.62 (0.96–2.72)	0.07
PNI								
Absent								
Present	1.57 (1.27–1.94)	<0.01	1.09 (0.87–1.36)	0.47	2.15 (1.33–3.46)	<0.01	2.06 (1.22–3.47)	0.01
CEA								
Negative								
Positive	1.73 (1.39–2.16)	<0.01	1.47 (1.16–1.85)	<0.01	1.89 (1.17–3.04)	0.01	2.25 (1.35–3.75)	<0.01
Neoadjuvant therapy								
No								
Yes	0.87 (0.56–1.34)	0.53			0.95 (0.35–2.60)	0.92		
Chemotherapy								
No								
Yes	0.57 (0.46–0.72)	<0.01	0.49 (0.39–0.62)	<0.01	1.11 (0.67–1.85)	0.69		
**LNR**		**<0.01**		**<0.01**		**0.01**		**0.01**
Low								
Moderate	2.22 (1.71–2.95)	<0.01	1.96 (1.37–2.79)	<0.01	1.72 (0.91–3.25)	0.10	1.77 (0.92–3.41)	0.09
High	3.96 (2.99–5.25)	<0.01	2.57 (1.78–3.72)	<0.01	2.79 (1.47–5.28)	<0.01	3.02 (1.54–5.92)	<0.01

HR, hazard ratios; CI, confidence intervals; TD, tumor deposit; PNI, perineural invasion; CEA, carcinoembryonic antigen; LNR, positive lymph node ratio.

**TABLE 4:** Univariate and multivariate analyses of overall survival in the two cohorts.

	The training cohort				The validation cohort			
	Univariate		Multivariate		Univariate		Multivariate	
	HR (95% CI)	*p value*	HR (95% CI)	*p value*	HR (95% CI)	*p value*	HR (95% CI)	*p value*
Gender								
Female								
Male	0.93 (0.76–1.14)	0.49			0.85 (0.54–1.34)	0.48		
**Age**	**1.00 (1.00–1.02)**	**0.01**	**1.01 (1.00–1.02)**	**<0.01**	**1.01 (1.00–1.03)**	**0.10**		
Tumor size								
≤5 cm								
>5 cm	1.44 (1.18–1.77)	<0.01	1.13 (0.92–1.40)	0.26	1.16 (0.75–1.79)	0.52		
**Tumor site**		**0.24**				**0.99**		
Right								
Left	0.93 (0.74–1.16)	0.93			0.99 (0.55–1.80)	0.98		
Rectum	0.72 (0.48–1.07)	0.10			1.01 (0.59–1.71)	0.98		
Grade								
Moderate								
Poor	1.20 (0.76–1.91)	0.43			2.11 (0.52–8.58)	0.30		
T stage								
T1-3								
T4	1.99 (1.61–2.45)	<0.01	1.49 (1.20–1.86)	<0.01	1.93 (1.22–3.04)	0.01	1.42 (0.89–2.27)	0.14
N stage								
<4 nodes								
≥4 nodes	2.31 (1.78–3.00)	<0.01	1.26 (0.88–1.80)	0.21	1.10 (0.66–1.84)	0.72		
M stage								
M0								
M1	2.29 (1.86–2.81)	<0.01	1.59 (1.27–2.00)	<0.01	2.11 (1.35–3.30)	<0.01	1.89 (1.15–3.09)	0.01
TD								
Absent								
Present	1.78 (1.46–2.19)	<0.01	1.35 (1.08–1.70)	0.01	2.57 (1.65–4.00)	<0.01	1.85 (1.14–3.00)	0.01
PNI								
Absent								
Present	1.53 (1.26–1.87)	<0.01	1.13 (0.91–1.40)	0.28	1.94 (1.23–3.04)	<0.01	1.81 (1.11–2.94)	0.02
CEA								
Negative								
Positive	1.69 (1.38–2.08)	<0.01	1.32 (1.05–1.65)	0.02	1.80 (1.15–2.81)	0.01	2.00 (1.26–3.20)	<0.01
Neoadjuvant therapy								
No								
Yes	0.80 (0.53–1.22)	0.30			0.81 (0.30–2.22)	0.69		
Chemotherapy								
No								
Yes	0.50 (0.41–0.62)	<0.01	0.48 (0.38–0.60)	<0.01	0.89 (0.56–1.40)	0.60		
**LNR**		**<0.01**		**<0.01**		**0.02**		**0.02**
Low								
Moderate	1.97 (1.54–2.53)	<0.01	1.81 (1.31–2.52)	<0.01	1.52 (0.85–2.70)	0.16	1.57 (0.87–2.84)	0.13
High	3.49 (2.69–4.52)	<0.01	2.51 (1.77–3.54)	<0.01	2.31 (1.29–4.14)	<0.01	2.42 (1.31–4.46)	0.01

HR, hazard ratios; CI, confidence intervals; TD, tumor deposit; PNI, perineural invasion; CEA, carcinoembryonic antigen; LNR, positive lymph node ratio.

**TABLE 5:** Multivariate analyses of cancer - specific and overall survival in two cohorts for the combined variable of TD, LNR, PNI.

	The training cohort				The validation cohort			
	Cancer-specific survival		Overall survival		Cancer-specific survival		Overall survival	
	HR (95% CI)	*p value*	HR (95% CI)	*p value*	HR (95% CI)	*p value*	HR (95% CI)	*p value*
Tumor size								
≤5 cm								
>5 cm	1.18 (0.94–1.48)	0.16	1.13 (0.91–1.4)	0.26	1.03 (0.64–1.66)	0.91	1.12 (0.71–1.77)	0.62
T stage								
T1-3								
T4	1.63 (1.29–2.05)	<0.01	1.56 (1.25–1.94)	<0.01	1.86 (1.10–3.14)	0.02	1.54 (0.96–2.48)	0.07
N stage								
<4 nodes								
≥4 nodes	1.86 (1.36–2.53)	<0.01	1.72 (1.30–2.27)	0.26	1.47 (0.78–2.78)	0.24	1.02 (0.59–1.76)	0.95
M stage								
M0								
M1	1.86 (1.48–2.35)	<0.01	1.73 (1.38–2.15)	<0.01	2.17 (1.34–3.53)	0.002	1.87 (1.18–2.97)	0.01
**CEA**								
Negative								
Positive	1.45 (1.15–1.83)	0.002	1.42 (1.14–1.76)	0.002	1.83 (1.11–3.01)	0.02	1.65 (1.04–2.62)	0.03
Chemotherapy								
No								
Yes	0.50 (0.39–0.63)	<0.01	0.44 (0.36–0.55)	<0.01	1.18 (0.70–2.00)	0.54	0.92 (0.57–1.47)	0.73
**Combined variable**		**<0.01**		**<0.01**		**0.001**		**0.002**
Triple-negative								
Intermediate	2.52 (1.48–4.29)	0.001	2.17 (1.38–3.41)	0.001	1.59 (0.64–3.94)	0.32	1.74 (0.79–3.84)	0.17
Triple-positive	3.05 (1.65–5.63)	0.001	2.77 (1.62–4.74)	0.001	5.12 (1.78–14.73)	0.002	5.03 (1.90–13.3)	0.001

HR, hazard ratios; CI, confidence intervals; TD, tumor deposit; PNI, perineural invasion; CEA, carcinoembryonic antigen; LNR, positive lymph node ratio; Triple-positive: high LNR, TD-positive, and PNI-positive, Triple-negative: low LNR, TD-negative, and PNI-negative, Intermediate: the remaining group.

### Construction and validation of the nomogram

**FIGURE 3:**
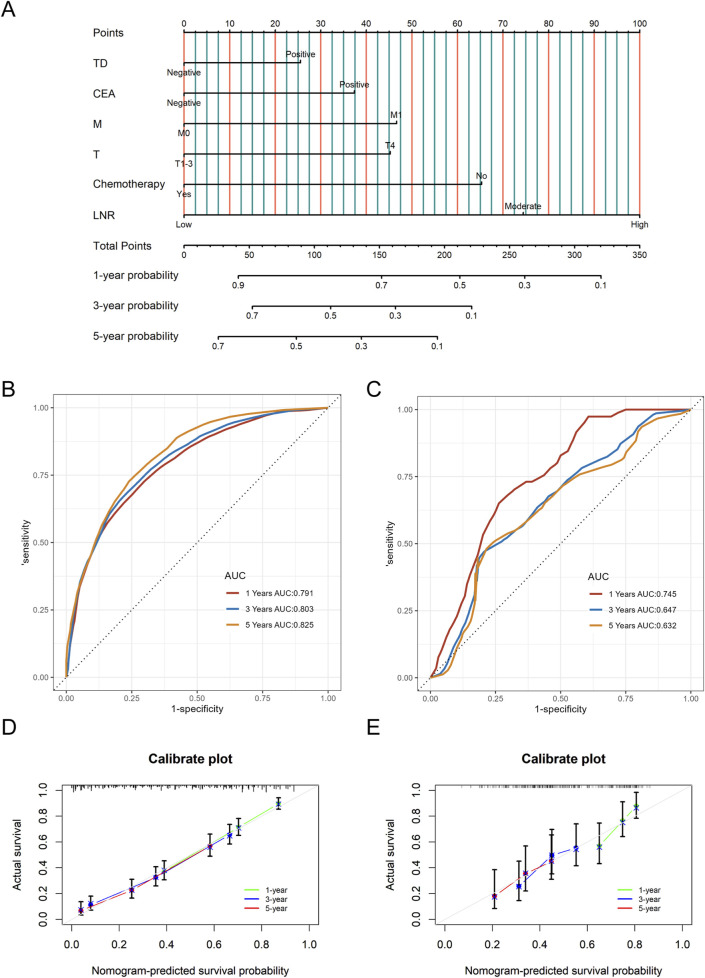
The nomogram model for CSS **(A)**, as well as time-dependent ROC curves **(B**, **C)** and calibration curves **(D**, **E)** in internal and external validation. CSS, Cancer-Specific Survival; LNR, positive lymph node ratio; TD, tumor deposit; ROC, receiver operating characteristic.

**FIGURE 4:**
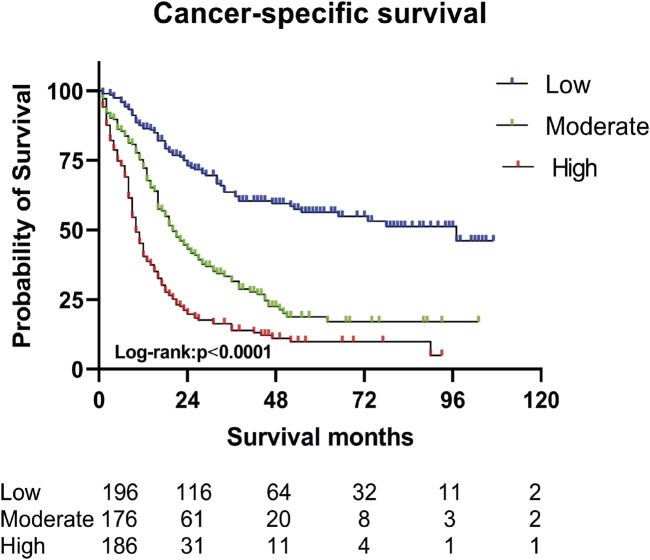
Survival curve analysis based on nomogram variable score groups (three risk groups).

**FIGURE 5:**
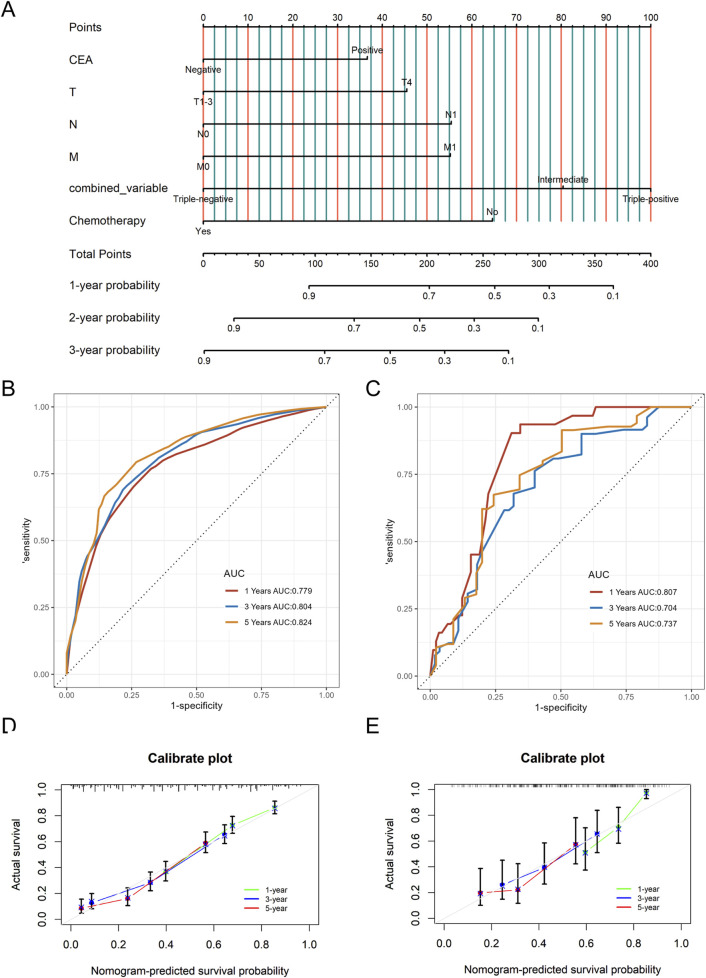
The nomogram model for CSS based on the combined variable of TD, LNR, and PNI **(A)**, as well as time-dependent ROC curves **(B, C)** and calibration curves **(D, E)** in internal and external validation. CSS, Cancer-Specific Survival; LNR, positive lymph node ratio; TD, tumor deposit; PNI, perineural invasion; ROC, receiver operating characteristic.

**FIGURE 6:**
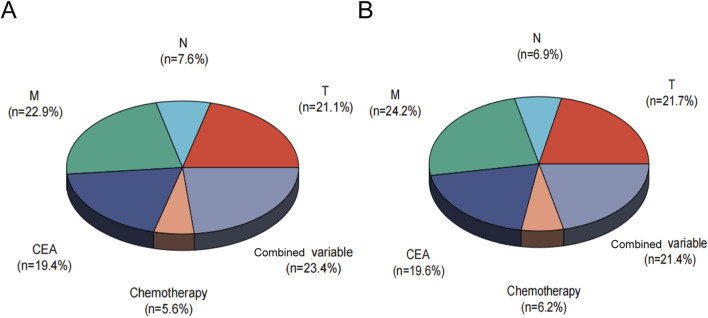
Relative contribution analysis of the combined variable for OS **(A)** and CSS **(B)**. CSS, Cancer-Specific; OS, Overall-Survival, Combined variable, the triple-positive group (high LNR, TD-positive, PNI-positive), triple-negative group (low LNR, TD-negative, PNI-negative), or intermediate group.

A nomogram predicting CSS in advanced colorectal SRCC was developed based on T stage, M stage, TD, CEA, chemotherapy, and LNR (Figure 3A). Internal validation using ROC curves showed AUC values of 0.79, 0.80, and 0.83 for 1-year, 3-year, and 5-year CSS, respectively (Figure 3B). In external validation, the corresponding AUCs were 0.75, 0.65, and 0.63 (Figure 3C). Calibration curves for 3-year and 5-year CSS closely aligned with the 45-degree diagonal line (Figures 3D,E). Patients stratified by nomogram scores into low-, moderate-, and high-risk groups exhibited significantly different survival outcomes on Kaplan-Meier analysis (Figure 4). A novel nomogram integrating the combined variable was constructed (Figure 5A). The model demonstrated favorable calibration and discriminatory ability, with internal validation AUCs of 0.78, 0.80, and 0.82, and external validation AUCs of 0.81, 0.70, and 0.74 for 1-, 3-, and 5-year CSS, respectively (Figures 5B–E). Relative contribution analysis revealed that the combined variable exhibited the highest relative contribution to OS at 23.4%, surpassing that of T and M stages. For CSS, its relative contribution was 21.4%, ranking second only to T and M stages (Figures 6A,B).

## Discussion

Signet ring cell carcinoma (SRCC) is a distinct subtype of adenocarcinoma, with remarkable differences from adenocarcinoma in pathological features, clinical behavior, prognosis, and other aspects (Dekker et al., 2019). SRCC originated primarily from mucosal epithelial cells. Microscopically, tumor cells displayed abundant mucin-filled cytoplasm, with over 50% signet ring cells in the tumor (Nagtegaal et al., 2020; Benesch and Mathieson, 2020). A higher proportion of signet ring cell components was associated with a worse prognosis (An et al., 2021; Song et al., 2019). In SRCC, the expression of adhesion molecules such as E-cadherin and β-catenin is downregulated, while intestinal trefoil factor and mucin two are upregulated, making it more prone to lymph node metastasis (Börger et al., 2007). Studies generally indicate that this type of tumor is insensitive to drugs such as cetuximab, oxaliplatin, and 5-fluorouracil (Pande et al., 2008). Therefore, exploring prognostic biomarker models related to colorectal SRCC for personalized treatment remains crucial.

LNR had demonstrated predictive value in gastrointestinal tumors (Zhu et al., 2018; Zhang C. et al., 2023). In the eighth edition of the UICC TNM classification, TD was defined as discrete nodules of cancer in the lymph drainage area of pericolorectal adipose tissue. TD was associated with poor prognosis in advanced stage rectal cancer (Wang et al., 2019). Previous studies indicated that the combined variable of TD and LNR had superior prognostic value for stage III CRC (Liu et al., 2024). PNI referred to the invasion of tumor cells into the nerve sheath and/or encirclement of the nerve circumference, which was associated with tumor invasion, metastasis, cancer-related pain, and poor clinical outcomes (Wang et al., 2024). Past studies documented the incidence of PNI across CRC stages: approximately 10% in stage I–II, 30% in stage III, and up to 40% in stage IV (Knijn et al., 2016; Al-Sukhni et al., 2017). However, the combined role of LNR, TD, and PNI as pathological biomarkers in advanced colorectal SRCC remained understudied.

The study found that TD was present in 50.7% of advanced colorectal SRCC cases, while PNI in 40.6% of cases. These rates were notably higher than those reported in general CRC literature (Knijn et al., 2016; Jin et al., 2015), likely due to the unique malignant pathological nature of SRCC. Moreover, high-LNR, TD-positive, and PNI-positive were all associated with poor prognosis. Further analysis revealed that LNR served as a consistent prognostic indicator across all stages, while TD and PNI showed significant prognostic value only in stage III, with no predictive significance in stage IV. This discrepancy may be attributed to the prevalence of distant metastasis in stage IV patients, which predominantly influences survival outcomes.

In this study, a nomogram model for advanced colorectal SRCC was constructed. By using individualized nomogram scores for risk stratification, the model effectively distinguished the survival outcomes of different subgroups, verifying its reliability. The combined analysis of TD, LNR, and PNI enhanced the prognostic assessment tool. The relative contribution analysis revealed that the combined variable accounted for 23.4% of OS prediction, surpassing the contributions of T and M stages. For CSS prediction, their combined weight reached 21.4%, second only to T and M stages. These findings underscored that TD, LNR, and PNI were essential biomarkers that complement the traditional staging system.

Nevertheless, the study has several limitations. As a retrospective design, it is inherently susceptible to selection and information biases. The absence of specific chemotherapy regimens and detailed patient health status limits the establishment of causal relationships. Secondly, the predictive performance of the nomogram diminishes over extended follow-up due to unmeasured confounding factors (e.g., secondary diseases, treatment changes). Future multicenter studies with larger sample sizes are necessary to enhance the model’s stability and generalizability.

In conclusion, LNR, TD, and PNI can serve as valuable prognostic factors for advanced colorectal SRCC. Their combined analysis represents a significant addition to the traditional staging system, offering a novel perspective for precise clinical prognosis assessment.

## Data Availability

The raw data supporting the conclusions of this article will be made available by the authors, without undue reservation.
