# Supplementary material for: Prognostic value of positive lymph node ratio, tumor deposit, and perineural invasion in advanced colorectal signet-ring cell carcinoma
Source: Front Mol Biosci. 2025 Aug 1;12:1617787. doi: 10.3389/fmolb.2025.1617787 (PMC12355033; doi:10.3389/fmolb.2025.1617787)
Supplement: Supplementary file 1 [file Supplementaryfile1.docx]

**Supplementary Fig 1** X-tile classified LNR into low-LNR, moderate-LNR, and high-LNR. LNR, positive lymph node ratio.


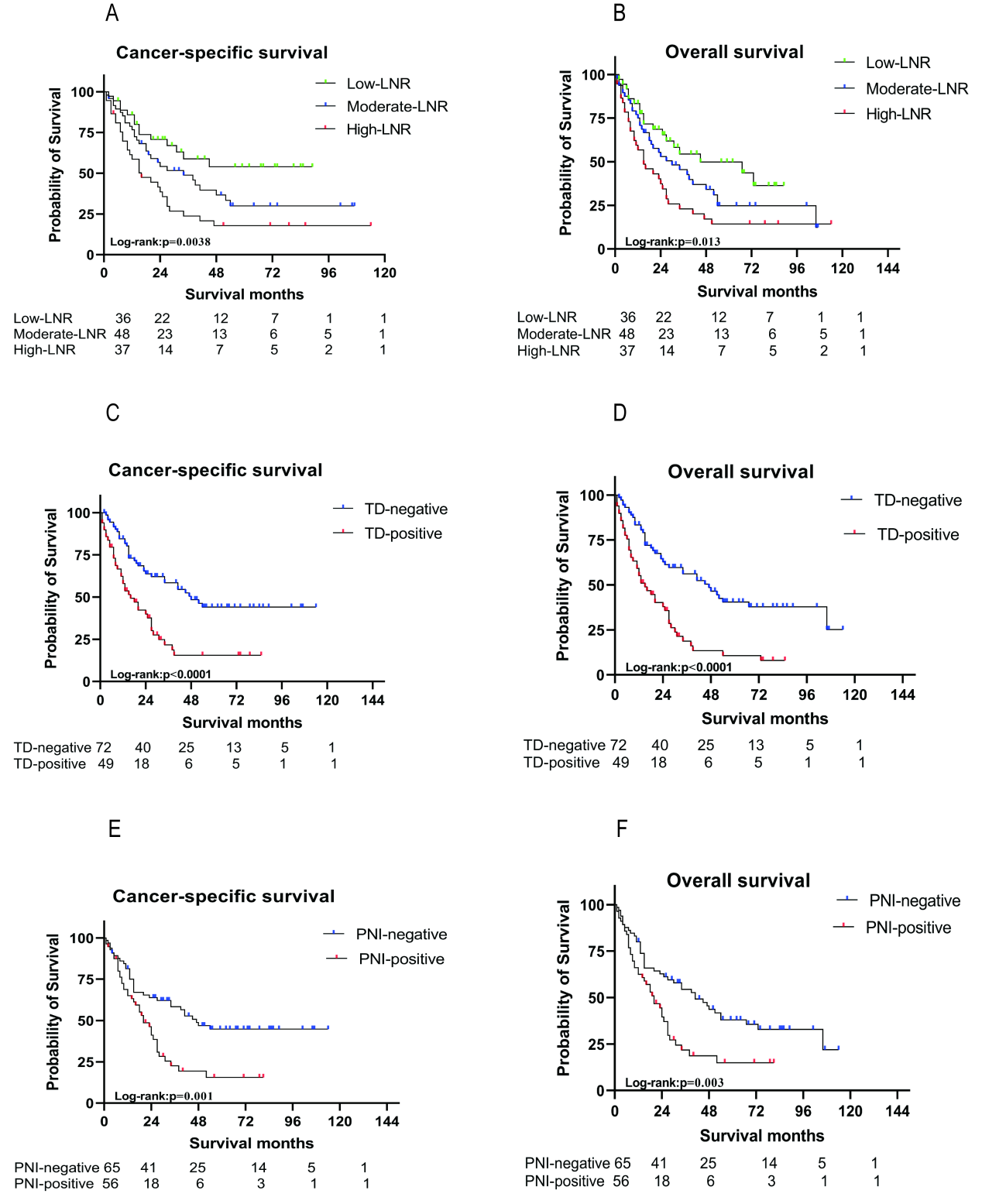


**Supplementary Fig 2.** In the validation cohort, Kaplan-Meier survival curves for CSS and OS based on LNR (A and B), TD (C and D), and PNI (E and F). OS, Overall Survival; CSS, Cancer-Specific Survival; LNR, positive lymph node ratio; TD, tumor deposit; PNI, perineural invasion.


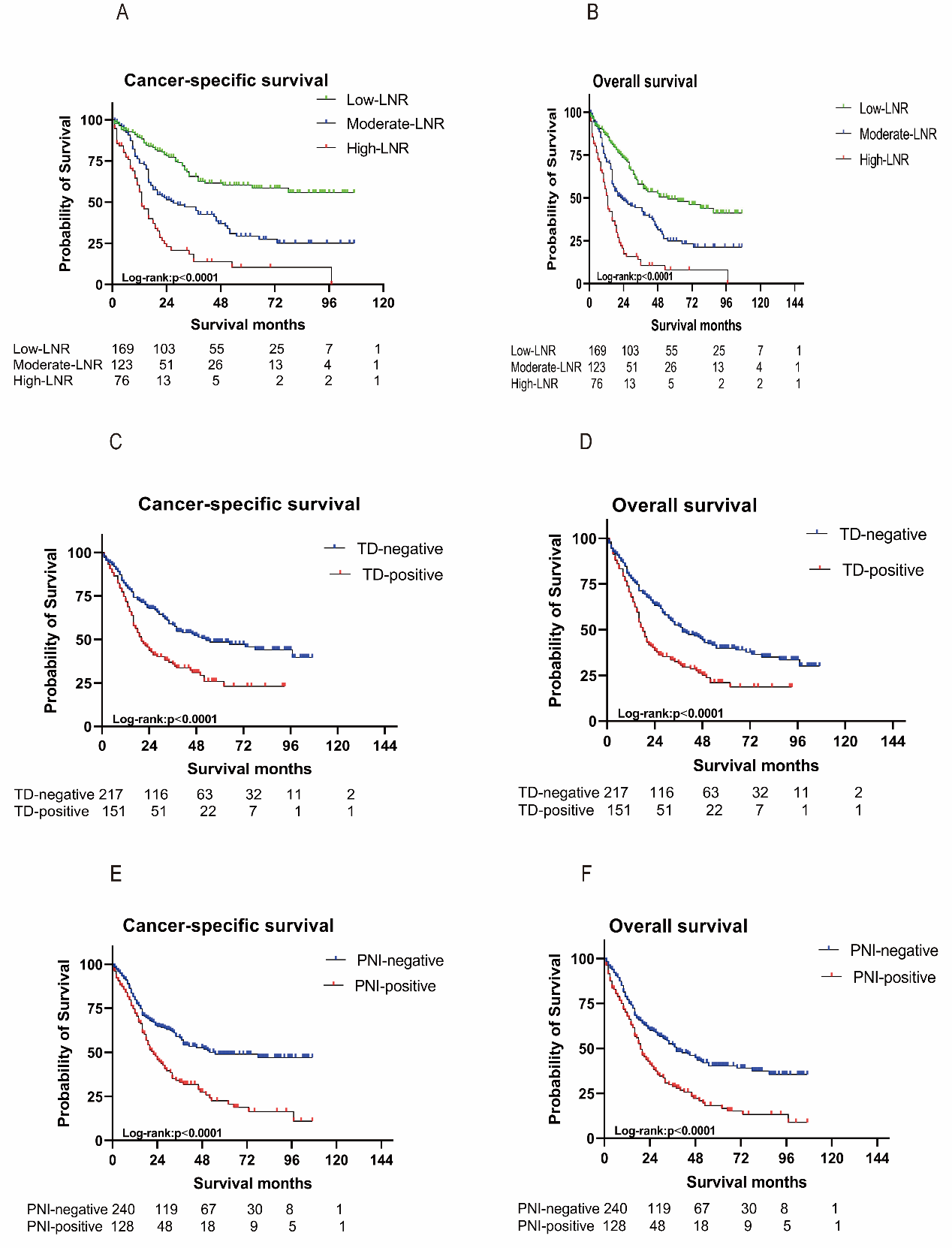


**Supplementary Fig 3.** In the training cohort, Kaplan-Meier survival curves for CSS and OS based on LNR (A and B), TD (C and D), and PNI (E and F) in stage III tumors. OS, Overall Survival; CSS, Cancer-Specific Survival; LNR, positive lymph node ratio; TD, tumor deposit; PNI, perineural invasion.


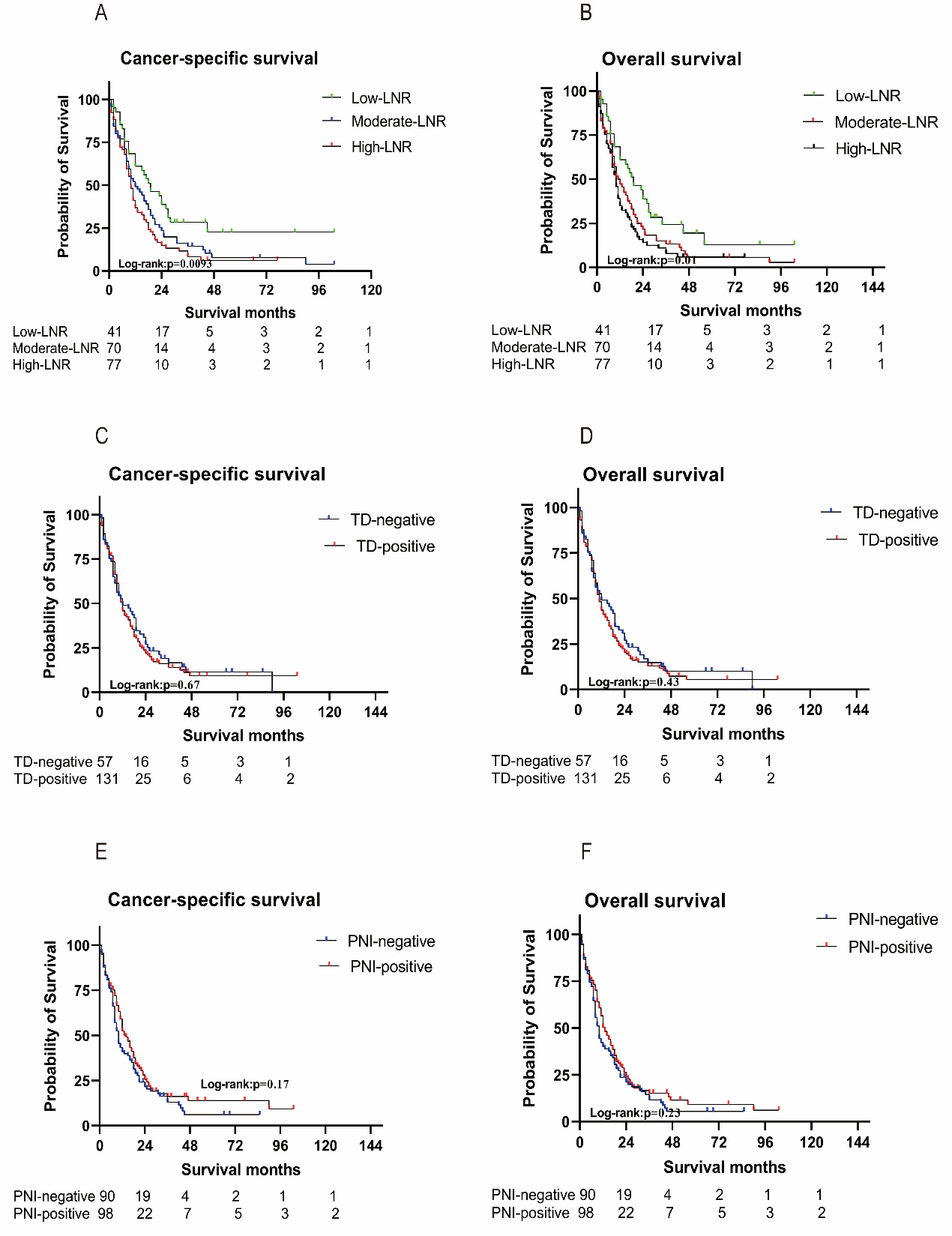


**Supplementary Fig 4**. In the training cohort, Kaplan-Meier survival curves for CSS and OS based on LNR (A and B), TD (C and D), and PNI (E and F) in stage IV tumors. OS, Overall Survival; CSS, Cancer-Specific Survival; LNR, positive lymph node ratio; TD, tumor deposit; PNI, perineural invasion.


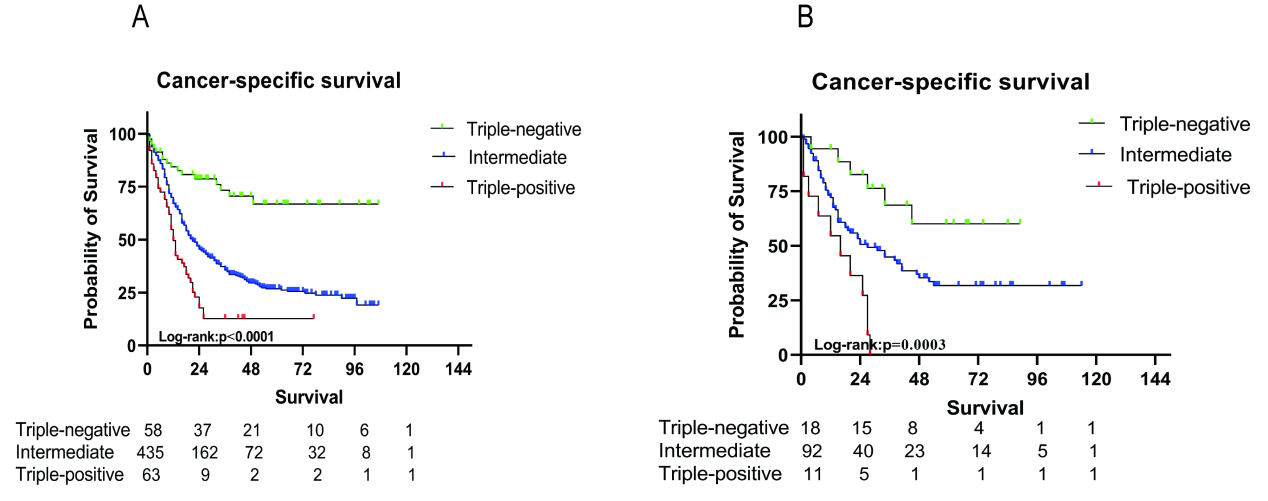


**Supplementary Fig 5.** Kaplan-Meier survival curves for CSS based on triple-positive, triple-negative and intermediate group in the training (A) and validation cohorts (B) .CSS, Cancer-Specific Survival.
